# Hypotensive and Hepatoprotective Properties of the Polysaccharide-Stabilized Foaming Composition Containing Hydrolysate of Whey Proteins

**DOI:** 10.3390/nu13031031

**Published:** 2021-03-23

**Authors:** Alexandr G. Kruchinin, Olga S. Savinova, Olga A. Glazunova, Konstantin V. Moiseenko, Evgeniya Yu. Agarkova, Tatyana V. Fedorova

**Affiliations:** 1 Federal State Budgetary Scientific Institution “All-Russian Research Institute of Dairy Industry”, 115093 Moscow, Russia; kruchinin-vnimi@yandex.ru (A.G.K.); euagarkova@mail.ru (E.Y.A.); 2 A.N. Bach Institute of Biochemistry, Research Center of Biotechnology of the Russian Academy of Sciences, 119071 Moscow, Russia; savinova_os@rambler.ru (O.S.S.); mr.moiseenko@gmail.com (K.V.M.)

**Keywords:** functional food, whey-protein hydrolysate, polysaccharides, foaming, spontaneously hypertensive rats (SHRs), angiotensin I-converting enzyme inhibition (ACE-I), antihypertensive effect, carbon tetrachloride-induced oxidative stress, hepatoprotection, TBARS

## Abstract

Whey protein hydrolysates (WPHs) are one of the most promising sources of biofunctional peptides with such beneficial properties as antioxidant, antihypertensive, anti-inflammatory and others. WPHs also could be used as foaming agents for aerated products (e.g., milk shake type drinks). However, WPH alone has a bitter taste and foamed WPH should be stabilized by additional ingredients. Here, we present a composition including WPH and three polysaccharides—pumpkin pectin, sodium alginate and ι-carrageenan—used as foam stabilizers. Polysaccharide content was selected according to foaming, organoleptic antioxidant and angiotensin-I-converting enzyme inhibitory characteristics of the resulted composition. Further, the hypotensive, antioxidant and hepatoprotective properties of the composition were proved by in vivo tests performed in spontaneously hypertensive rats and Wistar rats with CCl_4_-induced hepatic injury.

## 1. Introduction

A strong relation between food and health is known from the ancient times. Beyond doubt, a proper diet is the main risk-reducing factor for many chronic diseases, and understanding of the ways in which foods can offer health benefits beyond their nutritional value is fundamental issue of modern food science [1,2,3,4]. Nowadays, bioactive peptides are one of the most studied food compounds that may exert beneficial effects on a human health. Although biologically active peptides may be naturally released from their precursor proteins during digestion, this is a relatively inefficient process [5]. Thus, to obtain sufficient quantities of bioactive peptides from food sources, they usually have to be pretreated with a specifically designed mixture of proteases. Currently, an enormous number of sources for bioactive peptides as well as hydrolysis systems to release them have been reported [6].

For many years, hydrolysates of whey proteins (whey protein hydrolysates, WPHs) have been considered as an excellent nutritional source with a number of functional properties [7,8]. The functional properties of WPHs are mainly attributed to the biologically active peptides released in the process of hydrolysis of whey proteins by specific enzymes. Currently, such health-promoting properties of WPHs as antioxidant, antihypertensive, antithrombotic, anti-inflammatory, antimicrobial, anticancer and immunomodulatory have been reported [9,10,11]. Besides possessing functional properties WPHs have various technological advantages. Good swelling, solubility, gelling, water retention and fat binding capacities make whey proteins and their hydrolysates excellent food stabilizers [12,13]. Moreover, specific conditions of hydrolysis allow tailoring amount and size of peptides and altering such properties as gelation, foaming and emulsification abilities of WPHs [8]. As a result, whey proteins treated with limited hydrolysis could be used as foaming agents for aerated products [13,14].

Nowadays, different types of aerated products (e.g., whipped creams, mousses and milk-shake type drinks) are widely distributed in a global market [15]. In food systems, aeration allows getting an interesting pleasant consistency and texture, both of which generate a sensation of lightness and creaminess desirable to consumers. Possessing superior sensational characteristics, aerated products have a reduced cost of production and lowered calorific content [16]. The incorporation of protein hydrolysates can also significantly increase protein concentration in the final product, which is especially desired in production of sport-oriented protein-based supplementation.

In our previous work, we have found conditions of hydrolysis for whey proteins which allowed obtaining WPH with prominent antioxidant and angiotensin I-converting enzyme inhibitory (ACE-I) properties [17]. However, incorporation of this hydrolysate into food systems encountered two substantial hindrances. Firstly, the obtained WPH had a pronounced bitter taste. Unfortunately, as it was previously shown, bitterness is an inevitable side effect of functional properties for many hydrolysates [18,19]. This is especially true in the case of antioxidant and ACE-I properties. The main reason for hydrolysate bitterness is low molecular weight peptides containing hydrophobic amino acid residues [18,19]; however, the low molecular weight and hydrophobicity are the two general requirements for peptides to possess both antioxidant and ACE-I properties [20,21,22]. Secondly, although obtained WPH had a high foaming ability, the obtained foam had an insufficient stability.

It is well known that many plant polysaccharides can be used as foam-stabilizers in production of aerated drinks. As a hydrophilic high-molecular weight polymers, most polysaccharides do not adsorb at the air-water interface and enhance the stability of protein foams by acting as thickening or gelling agents [23]. In particular, such polysaccharides as ι-carrageenan, pectin and alginate found an especially wide application in technology. In addition to their gelling properties, these ingredients also exhibit prebiotic activity [24,25,26]. Moreover, for the pectin derived from pumpkin, the ability to mask the bitter taste of the hydrolysate was previously shown [27].

The aim of the current study was to develop a composition based on WPH and plant polysaccharides (WPH–PS composition) which possesses suitable foam-forming, organoleptic and functional properties and can be used for creating of functional aerated products.

## 2. Materials and Methods

### 2.1. Materials

Cheese whey from hard Montasio cheese was kindly provided by Sfoggiatech (Tradizioni Italiane, Moscow, Russia). The preparation of whey samples and enzymatic hydrolysis were conducted as described in [17]. Briefly, following conditions were used: Protamex (Novozymes A/S, Bagsværd, Denmark) and Alcalase (Novozymes A/S, Bagsværd, Denmark) in concentrations of 3% and 1% (*w*/*w*) of the protein content, 90 min reaction time, pH 7.0, and a temperature of 50 °C. The degree of hydrolysis was 12%. Protein content of WPH was 3.2% (*w*/*w*).

The process of extraction and characterization of pumpkin pectin was previously described in [28]. Alginate was purchased from Qingdao bright moon seaweed group Co. Ltd. (Qingdao, China) and ι-carrageenan Benvisco SI 100 was purchased from Shemberg (Mandaue City, Philippines). Other reagents used were of analytical grade, and Milli-Q water was used in all experiments.

### 2.2. Preparation and Characterization of Foams

Polysaccharides in a concentration of 0.25% (*w*/*w*) were added to WPH at 40 ± 2 °C and mixed using the Silent Crusher M homogenizer (Heidolph, Schwabach, Germany) at 15,000 rpm for 10 min. Mixed samples were heated up to 85 °C for 2 min and then cooled to 10 °C. Prior to any measurements, samples were kept at 4 °C for 24 h for gel formation. Foams were produced using BL350V mixer (Kocateq, Busan, South Korea), whipping speed was 10,000 rpm and whipping time was 2 min. Foaming capacity was defined as the ratio of the final volume to the initial volume of the mixture. Foam stability was evaluated by the time required to drain half the maximum volume.

The rheological characteristics of the mixtures were investigated using a DV-II + PRO viscometer (Brookfield Engineering, Middleboro, MA, USA) equipped with LV3 spindles. The measurements were carried out at 4 ± 2 °C for 60 s; the values were recorded every 10 s. The samples were tested at 100 rpm. All measurements were carried out in triplicate.

Zeta potential (electrokinetic potential) of WPH–PS compositions was measured using a Zetasizer Nano system (Malvern Instrument Ltd., Malvern, Worcestershire, UK) in a 1 mL cuvette at 25 °C.

The antioxidant activity of the WPH–PS compositions was determined by the ORAC (oxygen radical absorbance capacity) fluorescence method [29] using a Synergy 2 microplate photometer–fluorometer (BioTek, Winooski, VT, USA). The peroxyl radical was generated directly in the reaction medium during the thermal decomposition of the 2,2′-azobis (2-methylpropionamidine) dihydrochloride (AAPH, Sigma-Aldrich, St. Louis, MO, USA), initiated by incubation at 37 °C for 10 min according to [30]. The antioxidant activity was expressed as an amount of Trolox (Sigma-Aldrich, St. Louis, MO, USA) molar equivalents per g of protein: µmol(TE)·g^−1^(protein).

The in vitro ACE-I activity of the WPH–PS compositions was determined by their ability to inhibit ACE (Sigma-Aldrich, St. Louis, MO, USA). o-Aminobenzoyl-Phe-Arg-Lys(dinitrophenyl)-Pro (Sigma-Aldrich, St. Louis, MO, USA) was used as a substrate with internal fluorescence quenching [31]. Reaction was performed in a buffer containing 0.1 M Tris-HCl, 50 mM NaCl, 0.01 mM ZnCl_2_, pH 7.0. The measurements were carried out on a Synergy 2 microplate photometer-fluorometer (BioTek, Winooski, VT, USA). ACE-I properties of the samples were evaluated by determination of the IC_50_ concentration at which ACE activity decreased by 50%. IC_50_ was expressed as mg of protein per mL of sample: mg (protein)·mL^−1^.

All products were stored for 1 day at 5 °C before the sensory evaluation. The sensory evaluation was carried out using eight panelists familiar with the product. The samples were evaluated for consistency, flavor, taste, bitterness, color and overall acceptability, on a five-point hedonic scale. 

### 2.3. Animals and Ethical Approval

Hypotensive effect of selected WPH–PS composition was studied in 32-week-old male spontaneously hypertensive rats (SHRs) from Puschino Kennel of Laboratory Animals (Pushchino, Russia). The initial rats’ body weight was 312 ± 13 g, and the initial systolic (Psyst) and the diastolic (Pdiast) blood pressures were 186 ± 19 mmHg and 116 ± 22 mmHg, respectively. Hypocholysterolemic and hepatoprotective effects of WPH–PS composition were studied in 36-week-old male Wistar rats from the same breed from the vivarium of the Research Center of Biotechnology RAS (Moscow, Russia). At the beginning of the experiment, the rats’ body weight was 476 ± 63 g.

The animals were housed 4–5 per cage in controlled environment conditions (temperature 22 ± 1 °C and 12 h:12 h light-dark cycles). During the experiments, all rats consumed a standard diet (Laboratormkorm, Moscow, Russia). 12 h before the end of the experiment all feed residues were removed from the cells; upon the completion of the experiment, the experimental animals were placed in a chamber for carbon dioxide euthanasia (VetTech, Congleton, UK) for 3–5 min.

Experiments were conducted in accordance with the legislation on protection of animals used for scientific purposes in Russia (directive N199n dated 1 April 2016 of the Ministry of Health of Russia) and the EU (directive 1292010/63/EU). Experimental protocols were approved by the Ethics Committee for Animal Research of the Federal Research Center Fundamentals of Biotechnology of the Russian Academy of Sciences (#08 from 22.06.2020 and #17 from 26.10.2020).

### 2.4. Assay of Antihypertensive and Bifidogenic Effects in Spontaneously Hypertensive Rats (SHRs)

**Figure 1:**
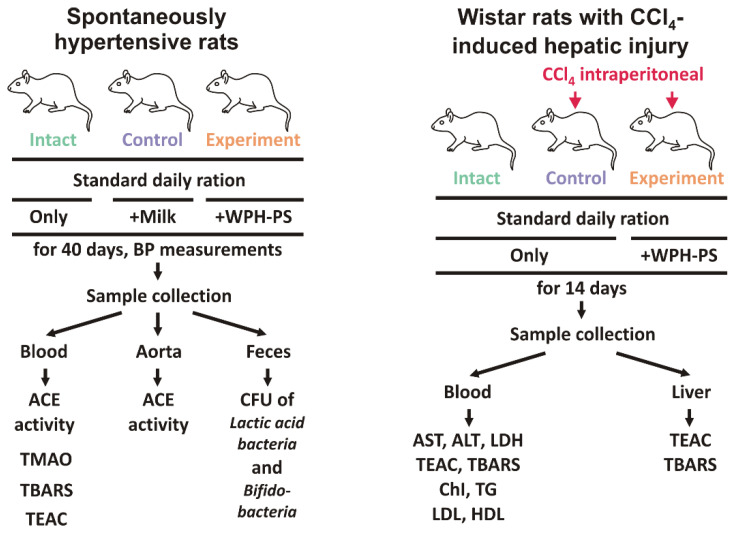
Experimental design.

For the experiment, the male SHRs were randomly divided into three groups of ten animals each: (1) receiving distilled water (the intact group); (2) receiving skim cow’s milk (the control group); (3) receiving WPH–PS composition: WPH and 0.25% of polysaccharides mixture consisting of alginate, ι-carrageenan and pumpkin pectin in the ratio of 2:3:1 (the experimental group). Experimental design is shown at Figure 1 (left panel).

The blood pressure of the rats was measured by the tail-cuff method with a CODA Monitor Rat-Cuff KIT (Kent Scientific, Torrington, CT, USA). At least ten measurement cycles were performed for each animal, and the results were averaged.

On the 40th day of the experiment, the animals were euthanized and blood, aorta and feces samples were collected. The blood serum was separated via centrifugation for 10 min with an Eppendorf 5702R centrifuge (Eppendorf, Hamburg, Germany) at 4 °C and 2000 g. ACE activity was measured in aorta and blood samples as described in paragraph 2.2 but without adding of ACE in the reaction mixture. Prior to measurements aorta samples were homogenized with Silent Crusher M homogenizer (Heidolph, Schwabach, Germany) in the buffer used for ACE activity measurements. To quantify the lactobacteria and bifidobacteria, rat feces were homogenized, and the inoculations were made into selective growth media for *Lactobacillus* spp. and *Bifidobacterium* spp. as described in [27]. Than the colony forming units (CFU) were counted.

### 2.5. Assay of Antioxidant and Hepatoprotective Effect in Wistar Rats with Carbon Tetrachloride-Induced Oxidative Stress

Oxidative stress was induced by intraperitoneal injection of CCl_4_ (0.5 mL per 1 kg of body weight) solution in refined deodorized sunflower oil (1:1 vol/vol) under diethyl ether anesthesia. Sunflower oil was bought in local market and sterilized via filtration through 0.22 μm PTFE syringe filter (Carl Roth, Karlsruhe, Germany).

For the experiment, the male Wistars were randomly divided into three groups of ten animals each: (1) receiving distilled water (the intact group); (2) receiving CCl_4_ and skim cow’s milk (the control group); (3) receiving CCl_4_ and WPH–PS composition: WPH and 0.25% of polysaccharide mixture consisting of alginate, ι-carrageenan and pumpkin pectin in ratio of 2:3:1 (the experimental group). Experimental design is shown at Figure 1 (right panel).

After 14 days of the experiment rats were euthanized; blood and liver were collected. The blood serum was separated via centrifugation for 10 min with 5702R centrifuge (Eppendorf, Hamburg, Germany) at 4 °C and 2000× *g*. For the analysis of antioxidant capacity, activity of liver enzymes and lipid profile, blood serum samples were frozen in liquid nitrogen. To analyze the content of thiobarbituric acid reactive substances (TBARS), 0.6 mL of blood serum was supplemented with 10 μL of 100 mg·mL^−1^ ionol solution in methanol as a stabilizer. Dissected liver samples were washed with chilled 0.15 M NaCl solution and the homogenates were prepared. All serum and liver samples were stored in a low-temperature (−86 °C) freezer prior to testing.

### 2.6. Blood Serum Biochemical Parameters

To determine the enzymatic activities of alanine aminotransferase (ALT), aspartic aminotransferase (AST) and lactate dehydrogenase (LDH), as well as to measure concentrations of total cholesterol (Chl), triglycerides (TG), low-density lipoproteins (LDL) and high-density lipoproteins (HDL), diagnostic kits for clinical chemistry (High Technology Inc., Attleboro Falls, MA, USA) were used. The analysis was performed on a BioChem FC-360 automatic chemistry analyzer (High Technology Inc., Attleboro Falls, MA, USA).

### 2.7. TMAO Analysis

Serum trimethylamine N-oxide (TMAO) concentration was determined as previously described by Wu et al. [32]. Serum samples were diluted in acetonitrile (1:10) and centrifuged at 16,000× *g* for 10 min (4 °C), and supernatants were collected and analyzed by UPLC-MS method using Agilent 6460C QQQ UPLC-MS/MS system (Agilent, Santa Clara, CA, USA). Chromatographic separation was performed using ACQUITY BEH HILIC 2 × 100 mm column (Waters, Worcester County, MA, USA). The flow rate of the mobile phase was 0.2 mL∙min^−1^. The mobile phase was (A) 0.1 mM formic acid water solution and (B) 0.1 mM formic acid solution in acetonitrile (LC-MS grade). Gradient condition was 85 to 65% B for 6 min, and the sample volume was 3 μL. The MS detector conditions were as follows: MS ESI(+); selected reaction monitoring, 76→59. An external standard including C16:0, C18:0 fatty acids (Supelco, Bellefonte, PA, USA) was used for quantitative analysis.

### 2.8. TEAC in Blood Serum and Liver Homogenates

The analysis of antioxidant capacity of blood serum and liver samples was performed using trolox equivalent antioxidant capacity (TEAC) assay with generation of ABTS radical as described in [33]. Before analysis, blood serum samples were thawed on ice and working dilutions were prepared in 75 mM sodium phosphate buffer, pH 7.4. Liver homogenates were prepared as follow: 100 mg of tissue were added to 4 mL of 11.5% NaCl solution and then homogenized for 3 min at 4 °C using Silent Crusher M homogenizer (Heidolph, Schwabach, Germany) operating at 75,000 rpm. For the blood, the antioxidant capacity of samples against ABTS radical was expressed as an amount of Trolox molar equivalents per L of sample: mM(TE); for the liver, the antioxidant capacity of samples against ABTS radical was expressed as an amount of Trolox molar equivalents per g of tissue: μmol(TE)·g^−1^(tissue).

### 2.9. TBARS Analysis

Lipid peroxidation was assessed by quantification of TBARS in liver homogenate and blood serum samples according to method of Jentzsch et al. [34] with minor modifications. For blood serum the reaction mixture contained 200 μL of sample, 30 μL of 54 mM of butylated hydroxytoluene (BHT) in methanol, 200 μL of 200 mM phosphoric acid and 200 μL of TBA reagent (0.11 M thiobarbituric acid solution in 0.1 M NaOH). For liver homogenate the reaction mixture contained 80 μL of sample, 20 μL of 54 mM BHT in methanol, 200 μL of 200 mM phosphoric acid, 40 μL of 8.1% SDS, 90 μL of ddH_2_O and 200 μL of TBA reagent. The reaction mixtures were incubated at 90 °C for 45 min in a water bath and then cooled to the room temperature. TBARS were extracted with 500 μL of 1-butanol and 50 μL of saturated NaCl solution and then the test tubes were centrifuged at 7000 rpm for 10 min using Mini Spin centrifuge (Eppendorf, Hamburg, Germany). Two hundred microliters of the upper butanol phase were taken for further analysis. Absorption was read at 535 nm and 572 nm using a Synergy 2 microplate photometer–fluorometer (BioTek, Winooski, VT, USA). Malondialdehyde (MDA) was used for calibration. For the blood, the results were expressed as an amount of MDA molar equivalents per L of sample: µM(MDA); for the liver, the results were expressed as an amount of MDA molar equivalents per g of tissue: nmol(MDA)·g^−1^(tissue).

For TBARS analysis liver homogenates were prepared as follow: 100 mg of tissue was added to homogenization buffer (1.15% KCl solution containing 2.0 mM ethylenediaminetetraacetic acid—EDTA and 2.5 mM ethylene glycol-bis(2-aminoethylether)-N,N,N′,N′-tetraacetic acid (EGTA), pH 7.4). Homogenization was carried out for 3 min at 4 °C using Silent Crusher M homogenizer (Heidolph, Schwabach, Germany) operating at 75,000 rpm.

## 3. Results and Discussion

### 3.1. Reological, Foam-Forming and Functional Properties of WPH–PS Compositions

At the first stage of the study, several polysaccharide compositions (samples 1–5, Table 1) containing different proportion of sodium alginate, ι-carrageenan and pumpkin pectin were tested as stabilizers for WPH foam. Although the proportion of sodium alginate, ι-carrageenan and pumpkin pectin (A:C:P) were different, the total polysaccharide content of all WPH–PS compositions was the same and comprised 0.25% (*w*/*w*). The main rheological and foam-forming properties of the obtained WPH–PS compositions are summarized in Table 1.

In general, the viscosity of the WPH–PS compositions was governed by the ratio of sodium alginate to pumpkin pectin. While an increase in the mass fraction of sodium alginate led to an increase in the dynamic viscosity for all samples, an increase in the mass fraction of pumpkin pectin produced the opposite effect. At the same time, the foam stability of the WPH–PS compositions was mainly linked to the proportion of ι-carrageenan. While the mass fraction of ι-carrageenan increased in series (sample 2 = sample 1 > sample 3 = sample 4 > sample 5), the foam stability increased in the series (sample 2 > sample 1 > sample 3 > sample 4 > sample 5). The foam capacity of the WPH–PS compositions was not strongly connected with the mass fraction of any particular polysaccharide, suggesting a presence of non-additive effects in their mixture.

In colloidal systems, the zeta potential is the electrical potential at the interface between immobilized and dynamic ionic layers of a particle suspended in a dispersion medium. Since the value of zeta potential determines the amount of electrostatic repulsion between particles, the zeta potential is intrinsically related to the stability of colloidal dispersions. However, the effect of the zeta potential on properties of foamed suspensions remains poorly explored [35]. In the current study, the foam stability of WPH–PS compositions generally increased with the increase of the absolute value of zeta potential. The highest stability was demonstrated by the samples 2 and 3 having zeta potential about −24 mV, while the sample 5 having zeta potential of −17.5 mV was the less stable. It should be noted that both samples 2 and 3 have a high proportion of sodium alginate and ι-carrageenan. This is in line with previous reports showing that the zeta potential of highly methoxylated pectins is significantly lower than that of alginates or carrageenans at all pH values, and during the electrostatic interaction of proteins with polysaccharides, the charge of the resulting complexes decreases [36,37,38].

**Figure 2:**
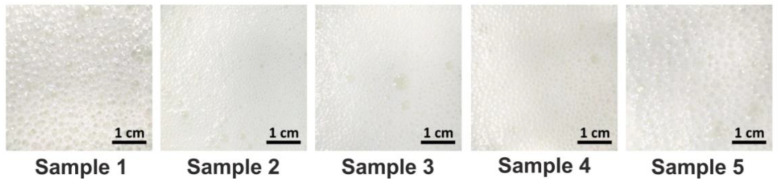
The bubble size and bubble homogeneity of WPH–PS compositions.

For all WPH–PS compositions, the quality of foams was evaluated in terms of bubble size, and bubble homogeneity (Figure 2). Bubble size increased in the series (sample 2 < sample 3 < sample 4 < sample 1 ≤ sample 5), and the size of the bubbles was negatively correlated with the bubble homogeneity. Generally, the samples with the higher pectin content were characterized by bigger bubble size. The smallest and the most homogeneous bubbles were observed in sample 2 (Figure 2).

The in vitro assessment of antioxidant and antihypertensive properties of the products are shown in Table 1. The antioxidant properties were measured by the ability of the samples to scavenge peroxyl radical (ORAC). The potential antihypertensive properties were assessed by the ability of the samples to inhibit ACE. Being a central component of the renin-angiotensin system, this enzyme controls blood pressure by regulating the volume of fluids in the body. In terms of biologically relevant differences, all the samples had comparable antioxidant properties around 400 µmol(TE)·g^−1^(protein). The greatest ACE-I activity was demonstrated by the sample 5, for which IC_50_ comprised 0.813 mg(protein)·mL^−1^.

**Figure 3:**
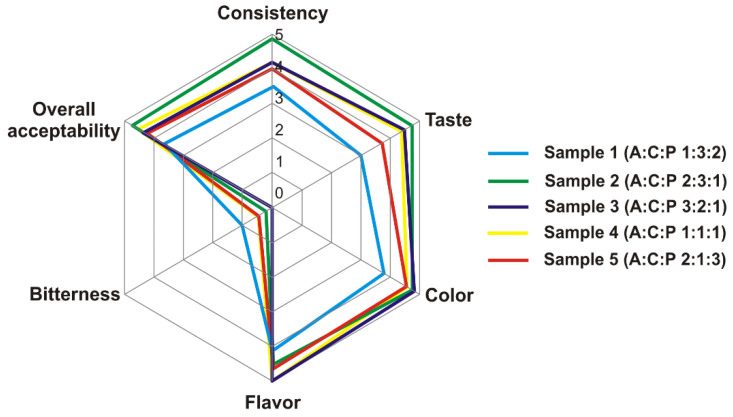
The organoleptic properties of WPH–PS compositions.

The organoleptic assessment of the samples is shown in Figure 3. All the studied WPH–PS compositions demonstrated a significantly decreased bitterness, compared to the original WPH (for which bitterness was assessed as 5). The entire absence of the bitterness was noted for the sample 3 and sample 4, while the sample 1 had the slightly bitter character. Although some experts assessed bitterness of sample 2 and sample 5 close to that of sample 1, the average bitterness of these samples was close to zero (i.e., entire absence). For the remaining organoleptic parameters, with a possible exception of flavor, the sample 2 outperformed all other WPH–PS compositions. Moreover, it was noted that the sample 2 had the most pronounced creamy mouthfeel. Hence, possessing high ORAC and ACE-I activities, sample 2 was characterized by the best organoleptic and foaming properties and was selected for the further in vivo trials.

### 3.2. The In Vivo Assessment of Antihypertensive Properties

The in vivo assessment of the antihypertensive properties for the sample 2 (further referred as just WPH–PS composition) was performed in SHR animal model, a popular animal model of essential (or primary) hypertension [39,40,41]. Around an age of 45 weeks, SHRs develop characteristics of cardiovascular disease such as abnormally increased blood pressure and vascular hypertrophy. Although several researches link the development of hypertension in SHRs with the malfunction of their kidneys, the exact molecular mechanisms are still to be discovered.

The experimental setup included three groups of ten SHRs: intact, control and experimental. While the intact animals received only a standard daily ration, the ration of the experimental group was supplemented with the WPH–PS composition. The SHRs which daily ration was supplemented with milk were used as a comparative control.

**Figure 4:**
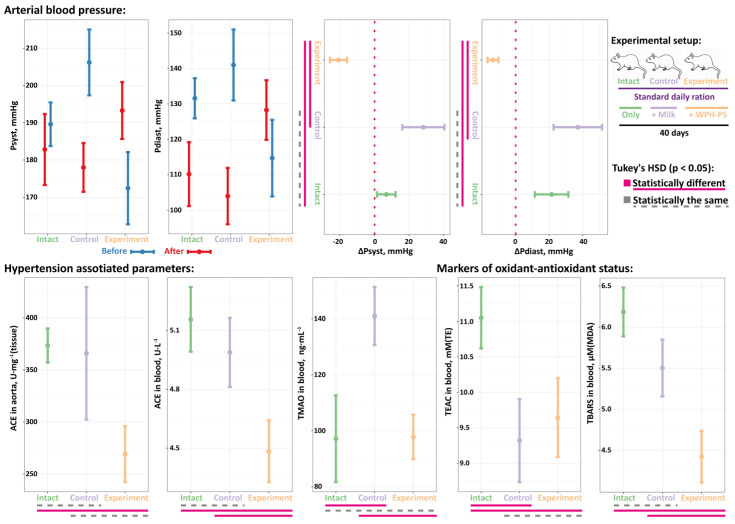
The in vivo assessment of antihypertensive properties for WPH–PS composition using Spontaneously Hypertensive Rats (SHRs) animal model. Each group of SHRs (intact, control and experimental) contained 10 animals.

The data on the absolute values and changes of the Psyst and Pdias for each animal group are present in Figure 4. For the intact and control animals, both Psyst and Pdias increased at the end of the experiment. In contrast, a significant decrease of both Psyst and Pdias was observed in the experimental group. Although ΔPsyst and ΔPdiast were not statistically significantly different for the intact and control animals, there was a tendency for control group to have a greater increase in blood pressure (i.e., greater averaged ΔPsyst and ΔPdiast) compared to the intact group.

For all animal groups, two parameters previously associated with the development of hypertension were measured—the activity of ACE both in aorta and blood and the concentration of TMAO in the blood (Figure 4). In the case of SHRs, the abnormal activity of ACE can be seen as an internal to the organism factor [42]. In contrast, the level of TMAO is highly related to the diet and condition of gut microbiome, since TMAO is generated from the oxidation of trimethylamine (TMA) produced by the gut microbiota [43]. TMA can be synthesized by the intestinal microflora from choline, L-carnitine and betaine contained in food. Upon its transportation to the liver, TMA is oxidized into TMAO by liver enzyme—flavin-containing monooxygenase 3 (FMO3). Elevated concentration of TMAO in the blood leads to inactivation of endothelial nitric oxide synthase (eNOS) and activation of oxidative stress, which lead to endothelial dysfunction and triggers the processes of atherosclerosis and vasoconstriction [43].

For the ACE activity, its values both in the aortas and blood of the experimental animals were significantly lower compared to the intact group; for the intact and control animals, the ACE activity was statistically the same in the aortas and blood, respectively. While the values of TMAO for the intact and experimental animals were similar, its value in the control group was significantly increased. The follow-up study of the animal feces demonstrated a prominent bifidogenic effect of the WPH–PS composition: the amount of *Bifidobacterium* spp. in the feces of the experimental animals, (1.0–1.4)·10^9^ CFU·g^−1^(feces), was significantly higher than that in the feces of intact and control animals, (2.0–2.5)·10^8^ CFU·g^−1^(feces). At the same time, the amount of *Lactobacillus* spp. in all groups of animals was the same and comprised (1.1–1.2)·10^6^ CFU·g^−1^(feces).

The level of the oxidative stress in all studied animals was assessed by measuring TEAC and TBARS in the animals’ blood (Figure 4). The TEAC value represent an overall antioxidant capacity of biological sample which can be increased as a result of an oxidative stress [44], and TBARS value corresponds to the advancement of lipid peroxidation under the oxidative stress. The level of TEAC in the blood of the intact animals was significantly higher compared to the control and experimental groups, for which no significant differences were observed. For the level of TBARS, while it was statistically the same in the intact and control groups, in the experimental animals it was significantly lowered.

Hence, the experiments with SHR animal model demonstrated that WPH–PS composition possess antihypertensive properties in vivo. The lowering of systolic and diastolic blood pressure in SHRs upon the administration of the WPH–PS composition is most likely related to the inhibition of ACE, which is in compliance with the assessment of WPH–PS composition’s functional properties in vitro. Additionally, it was demonstrated that the administration of the product did not significantly increase level of TMAO; therefore, the product did not alter the composition of gut microbiota in a harmful direction. Moreover, the follow-up study demonstrated a prominent bifidogenic effect of the WPH–PS composition. Earlier it was shown that high protein diet can increase level of TMAO [45]. However, in our study we demonstrated that consumption of hydrolyzed milk proteins as a part of WPH–PS composition does not cause, in contrast to milk, increase in the level of TMAO, which is possibly partly associated with protein pre-hydrolysis and the bifidogenic effect of WPH–PS composition.

Previously it was demonstrated that although oxidative stress does not causatively relate to the development of hypertension in SHRs, in these rats the oxidative damage is progressing slowly as a consequence of hypertension [44]. In the current experiment, the decreased levels of TEAC and TBARS in the experimental animals suggested that administration of the WPH–PS composition could partially relieve the oxidative stress. Most probably, this effect can be attributed to the antioxidant peptides presence of which was previously determined in the WPH used in the current work by in vitro assessment [17].

### 3.3. The In Vivo Assessment of Hepatoprotective Properties

**Figure 5:**
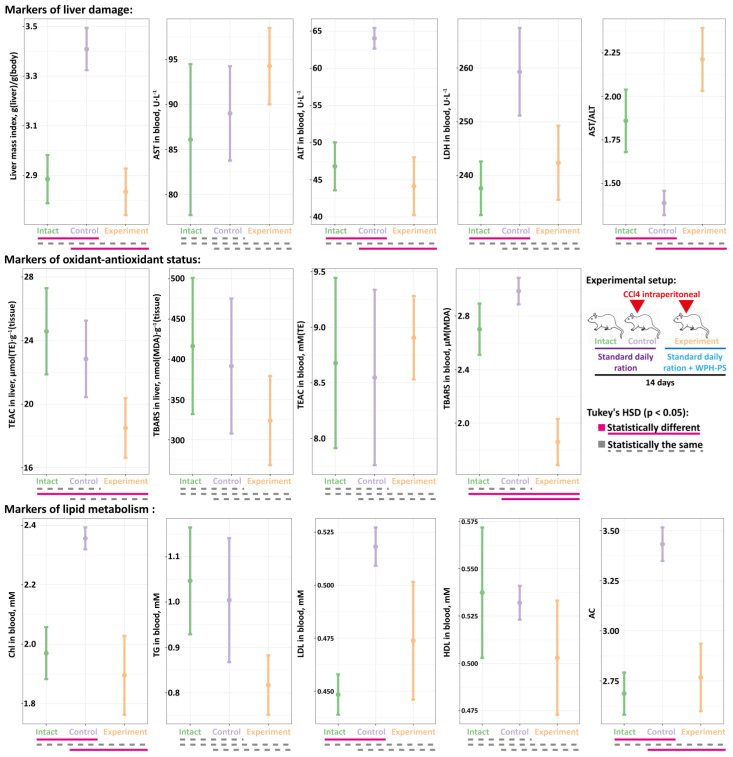
The in vivo assessment of hepatoprotective properties for WPH–PS composition using Wistar rats with CCl_4_-induced hepatic injury animal model. Each group of Wistars (intact, control and experimental) contained 10 animals.

Based on the determined in vitro and in vivo antioxidant activity of the WPH–PS composition it was proposed that its administration can alleviate effects of an oxidative stress to an organism. It is well known that a toxic dose of CCl_4_ increases the concentration of reactive oxygen species (ROS) in the liver causing acute liver damage [46,47,48]. Hence, the in vivo antioxidant property of WPH–PS composition was assessed using a rat model with CCl_4_-induced hepatic injury (Figure 5). The hepatotoxicity was induced by the administration of a single intraperitoneal dose of CCl_4_. Thereafter, a standard daily ration of an experimental group of rats was supplemented with WPH–PS composition, while the ration of a control group was unaltered. Additionally, a group of rats without CCl_4_-induced hepatic injury on the standard ration was used as an intact control.

To assess liver damage, the direct measurement of a liver to body weight ratio (liver mass index, LMI) was performed, and standard blood biomarkers of liver injury (i.e., AST, ALT and LDH) were measured (Figure 5). As it was expected, the control group demonstrated the consequences of the acute liver damage manifesting themselves in the statistically significant increase of LMI, ALT and LDH with concomitant decrease of AST to ALT ratio (AST/ALT) compared to the intact animals. For the experimental group, values of both LMI and blood biomarkers were the same as for the intact animals.

The oxidant-antioxidant status of the liver was assessed by measurement of TEAC and TBARS both in the liver homogenate and in blood serum (Figure 5). The values of TBARS in the liver homogenate as well as the values of TEAC in the blood were not statistically significantly different for all studied groups. The level of TEAC in the liver of experimental animals was significantly lower compared to the intact and control groups, for which it was the same. Although no statistically significant difference was detected between TBARS in the blood of intact and control groups, there was a tendency for the control animals to have higher TBARS level. The level of TBARS in the blood of the experimental animals was significantly lower than that in the control and intact groups.

Since liver is a central organ for metabolism of lipids, for all animal groups, such blood biomarkers as concentrations of Chl, TG, LDL and HDL were measured, and the value of atherogenic coefficient (AC) was calculated according to Nimmanapalli et al. [49] (Figure 5). There was no significant difference detected in the levels of TG and HDL among studied groups. While the level of LDL for the control group was significantly higher than that for the intact animals, the level of LDL for the experimental group was not statistically different from that in other two groups. The levels of Chl and AC for the control group were significantly higher compared to the intact and experimental animals. For the latter two groups, the levels of Chl and AC were the same.

Hence, the experiments on the rat model with CCl_4_-induced hepatic injury demonstrated that the administration of the WPH–PS composition can alleviate the consequences of the acute liver damage. Presumably, the antioxidant peptides present in the WPH–PS composition can participate in the quenching of highly reactive free radicals that are formed in the rats’ liver during CCl_4_ biotransformation through the cytochrome P-450 system. The early quenching of these radicals mitigates the following oxidative stress, oxidative degradation of lipids and degree of inflammation [50,51].

## 4. Conclusions

Usually, functional and technological properties of food products are studied separately; however, it is extremely important that useful functional components can be included in a product without deterioration of its technological and consumption features. In this article the aerated system containing WPH and mixture of polysaccharides (i.e., sodium alginate, ι-carrageenan and pumpkin pectin) was developed. As was demonstrated in vitro, the obtained WPH–PS composition retained antioxidant and ACE-I properties of the original WPH, while its bitterness and foam instability were successfully mitigated. Moreover, the performed in vivo studies using SHRs and Wistar-rats with CCl_4_-induced hepatic injury animal models demonstrated that systematic consumption of the developed WPH–PS composition exert beneficial health effects on an organism. The experiments with SHRs demonstrated that WPH–PS composition possess antihypertensive properties (i.e., lower Psyst and Pdias due to the inhibition of ACE) and did not alter gut microbiota in a harmful direction (i.e., increased production of TMA). Additionally, consumption of WPH–PS composition decreased levels of TEAC and TBARS in the blood of animals suggesting alleviation of oxidative stress. The experiments with Wistar-rats animal model demonstrated that WPH–PS composition possesses hepatoprotective properties attenuating the consequences of the acute liver damage caused by ROS.

## Figures and Tables

**Table 1:** The rheological, foam-forming and functional properties of compositions comtaining whey protein hydrolysates and plant polysaccharides (WPH–PS).

Properties	Polysaccharide Ratio				
	A:C:P * 1:3:2	A:C:P 2:3:1	A:C:P 3:2:1	A:C:P 1:1:1	A:C:P 2:1:3
	Sample 1	Sample 2	Sample 3	Sample 4	Sample 5
Viscosity, mPa·s	158	183	338	146	74
Foam capacity, %	280	400	340	360	380
Foam stability, min	41	48	35	27	15
Zeta potential, mV	−20.1	−23.7	−24.5	−20.1	−17.5
ORAC, µmol(TE)·g^−1^(protein)	418.3	393.4	399.3	405.4	407.9
ACE-I activity (IC_50_), mg(protein)·mL^−1^	1.66	1.07	1.27	1.01	0.813

* A—sodium alginate, C—ι-carrageenan, P—pumpkin pectin.

## Data Availability

Data is contained within the article or supplementary material.
